# Physical activity and alexithymia: chain mediation of sense of coherence and social anxiety—a study of chemistry majors

**DOI:** 10.3389/fpsyg.2026.1853496

**Published:** 2026-06-24

**Authors:** Yang Lv, Yang Liu

**Affiliations:** 1 College of Environmental and Chemical Engineering, Dalian University, Dalian, China; 2 College of Physical Education, Jilin University, Changchun, China

**Keywords:** alexithymia, chain mediation, physical activity, sense of coherence, social anxiety

## Abstract

**Aim:**

This study aimed to explore the effect of physical activity on alexithymia and to establish a chain mediation model with sense of coherence and social anxiety as mediators. A cross-sectional design was adopted.

**Methods:**

A total of 650 chemistry major students from six universities in the eastern coastal region of China were surveyed using the Physical Activity Rating Scale, the Toronto Alexithymia Scale, the Sense of Coherence Scale, and the Social Anxiety Scale.

**Results:**

(1) Physical activity was negatively correlated with alexithymia, although the magnitude of this correlation was small (*r* = −0.088, *P* < 0.01). Sense of coherence was negatively correlated with alexithymia (*r* = −0.473, *P* < 0.01). Social anxiety was positively correlated with alexithymia (*r* = 0.664, *P* < 0.01). Physical activity was positively correlated with sense of coherence (*r* = 0.101, *P* < 0.05) and negatively correlated with social anxiety (*r* = −0.119, *P* < 0.01), both with small effect sizes. Sense of coherence was negatively correlated with social anxiety (*r* = −0.430, *P* < 0.01). (2) Physical activity had a negative predictive effect on alexithymia among university students, with a direct effect value of −0.200. (3) Sense of coherence and social anxiety not only played separate mediating roles but also a chain mediating role between physical activity and alexithymia, with effect sizes accounting for 7.06, 24.12, and 15.88% of the total effect, respectively.

**Conclusion:**

Physical activity shows a statistically significant but weak negative association with alexithymia among chemistry major students (*r* = −0.088, small effect size). The direct protective effect is limited. However, physical activity influences alexithymia indirectly through the separate mediating effects of sense of coherence and social anxiety, as well as through their chain mediating effect, which together account for a substantial portion (47.06%) of the total effect. Due to the cross-sectional design, causal relationships cannot be inferred from these findings; the results primarily indicate associations and mediating pathways that require longitudinal or experimental validation.

## 1 Introductory

Alexithymia refers to a personality trait characterized by significant difficulties in identifying and describing one’s own emotions (Lu and Li, 2026). It manifests as difficulty distinguishing between emotions and bodily sensations, lack of imagination, and an externally oriented thinking style. Although not an independent mental disorder, alexithymia is a potential risk factor for various psychosomatic diseases and emotional disorders (Li et al., 2022). Its formation involves both cognitive deficits (e.g., incomplete emotional schemas, insufficient theory of mind, executive dysfunction) and social factors (e.g., childhood environment, family functioning, sociocultural context) (Zhang et al., 2011). Individuals with alexithymia often struggle to identify and express emotions, sometimes replacing genuine feelings with “protective” emotions like anger, which worsens daily functioning. This risk may be particularly high among chemistry majors. Recent empirical studies indicate that first-year chemistry students report alarming rates of moderate to severe depression (49.3%) and anxiety (56.6%) (Pfau, 2025), and that STEM students experience more severe depressive symptoms and higher levels of alexithymia than their non-STEM peers (Zhang et al., 2024). Moreover, the chemistry laboratory environment can induce sensory overload prolonged exposure to strong lighting, chemical odors, protective gear, and sustained noise which negatively affects students’ emotional states (Stone and Arenas, 2025). Compounding this, the emotionally demanding nature of laboratory work and the relative lack of humanistic care in science education may further inhibit emotional expression (Jahanaray et al., 2025). Physical activity activates the central nervous system and regulates emotions via neurotransmitter release. For individuals with alexithymia, physical activity may accelerate emotion classification, increase sensitivity to emotional stimuli, and exert a beneficial regulatory effect (Li, 2020). However, important gaps remain: although physical activity benefits emotional processing in general populations, its relationship with alexithymia among chemistry majors has not been empirically examined. Furthermore, while sense of coherence and social anxiety are individually associated with alexithymia, it is unknown whether they serve as sequential (chain) mediators in the link between physical activity and alexithymia. Existing research also tends to focus on direct effects, overlooking the complex psychological pathways through which physical activity may indirectly reduce alexithymia. Accordingly, the present study aims to investigate the effect of physical activity on alexithymia among chemistry majors and to examine the chain mediating role of sense of coherence and social anxiety, thereby providing a theoretical basis for targeted emotional health interventions.

## 2 Research hypothesis

### 2.1 Prediction of the effect of physical activity on alexithymia among chemistry major students

In this study, physical activity refers specifically to planned, structured, and repetitive bodily movements undertaken to enhance physical fitness and health—a concept that aligns with the World Health Organization’s definition of *exercise* (a subcategory of physical activity) (World Health Organization, 2021). Unlike daily physical movement, physical activity emphasizes goal orientation and regularity, typically involving specific intensity, duration, and frequency (Qiao, 2025). As a positive lifestyle intervention, the promoting effect of physical activity on individual physical and mental health has been confirmed by a large body of research. From a physiological mechanism perspective, physical activity can influence individuals’ emotional processing through multiple pathways. Regular exercise promotes the release of brain-derived neurotrophic factor (BDNF), which contributes to the functional maintenance of emotion-related brain regions such as the hippocampus. Meanwhile, exercise also regulates the secretion levels of neurotransmitters such as dopamine and serotonin, which are directly involved in emotional regulation and stress coping processes (Tao, 2023). From a psychological mechanism perspective, physical activity provides individuals with an outlet for emotional catharsis. By diverting attention and improving self-perception, it helps individuals establish more positive emotional experiences (Chen and Qian, 2025). Therefore, physical activity may not directly improve the emotion recognition ability of individuals with alexithymia, but it may enhance their emotional processing efficiency, especially by increasing sensitivity to emotions. This may have important intervention value for individuals with alexithymia characterized by emotional blunting. Based on this, we propose Hypothesis H1: Physical activity is negatively associated with alexithymia among chemistry major students.

### 2.2 Prediction of the mediating effect of sense of coherence

The concept of sense of coherence was proposed by Antonovsky, it refers to an individual’s global orientation toward life as comprehensible, manageable, and meaningful. Sense of coherence is considered a stable psychological resource that helps individuals cope with stress and maintain health (Yin, n.d.). Critically, sense of coherence is not static; it can be shaped by repeated experiences that generate consistency, participation, and favorable outcome appraisal. Physical activity provides such experiences: engaging in regular, structured exercise yields frequent success experiences, positive feedback, and a sense of accomplishment. Consequently, physical activity is theorized to enhance sense of coherence over time (Yu and Yin, 2017). This directional logic—physical activity → increased sense of coherence—is supported by cross-sectional evidence that favorable physical activity attitudes correlate positively with sense of coherence (Yu and Yin, 2017), but more importantly, by the intrinsic properties of exercise as a mastery experience. In turn, sense of coherence is hypothesized to influence alexithymia. Individuals with a strong sense of coherence view internal and external stimuli as structured, manageable, and worthy of engagement. This cognitive orientation fosters attentional vigilance toward emotional information and facilitates the development of emotional schemas. Conversely, low sense of coherence is associated with negative emotional self-schemas and avoidance of positive emotional cues, which are core features of alexithymia (Hu, 2013). Thus, sense of coherence likely acts as a protective factor that reduces alexithymia by improving emotion recognition and expression. Integrating these two directed relationships, we propose that sense of coherence mediates the effect of physical activity on alexithymia: physical activity enhances sense of coherence, which in turn reduces alexithymia. This mediational pathway is theoretically grounded in the stress-resource model and empirical studies linking exercise to psychological resources and emotional processing (Hu, 2013). Based on this, we propose Hypothesis H2: Sense of coherence mediates the relationship between physical activity and alexithymia among chemistry major students. Specifically, physical activity is positively associated with sense of coherence, and sense of coherence is negatively associated with alexithymia.

### 2.3 Prediction of the mediating effect of social anxiety

Social anxiety refers to a significant and persistent fear experienced by individuals in real or imagined social situations due to concerns about being negatively evaluated by others (Rose and Tadi, 2022). While this condition can reach clinical severity, in non-clinical populations it is best understood as a continuous dimension ranging from mild discomfort to more pronounced distress that may interfere with daily functioning. In the present non-clinical sample of university students, social anxiety is operationalized as the level of self-reported anxious distress in social interactions, as measured by the Interaction Anxiousness Scale (IAS) (Jefferson, 2001). This approach avoids over-pathologizing and aligns with research on student mental health, where sub-clinical social anxiety is common and functionally relevant. The alleviating effect of physical activity on social anxiety has received substantial research support, with the frequency and duration of physical activity being significantly negatively correlated with the level of social anxiety (Cheng et al., 2025). From the perspective of underlying mechanisms, physical activity may indirectly reduce social anxiety by improving emotion regulation abilities: regular exercise helps individuals more effectively employ adaptive emotion regulation strategies such as cognitive reappraisal and reduces emotional suppression responses, thereby enabling them to maintain greater emotional stability when facing social situations (Hui et al., 2025). Regarding the relationship between social anxiety and alexithymia, the core characteristic of alexithymia—difficulty in identifying and describing one’s own emotions—constitutes an important cognitive basis for social anxiety. Studies have confirmed that individuals with alexithymia, due to their difficulty in accepting and understanding their own emotions, experience more distress in social interactions, which in turn reinforces social avoidance tendencies (Edel et al., 2010). Thus, social anxiety is not only an emotional correlate of alexithymia but may also, in turn, exacerbate difficulties in emotion recognition. Based on this, we propose Hypothesis H3: Social anxiety mediates the negative association between physical activity and alexithymia among chemistry major students. Specifically, physical activity is negatively associated with social anxiety, and social anxiety is positively associated with alexithymia.

### 2.4 Prediction of the chain mediating role of sense of coherence and social anxiety

**FIGURE 1:**
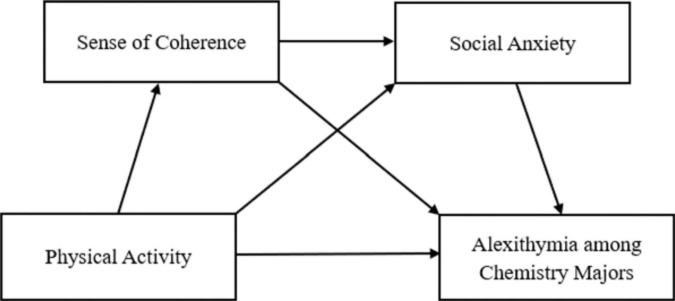
Diagram of the hypothetical model.

A close negative association exists between sense of coherence and social anxiety. As a core psychological resource for coping with stress, sense of coherence determines how individuals perceive and respond to challenges from the environment (Ge et al., 2019). Individuals with a high sense of coherence tend to perceive social situations as comprehensible, manageable, and meaningful, which reduces threat appraisals and lowers the likelihood of triggering social anxiety (da-Silva-Domingues et al., 2025). This logic aligns with the conservation of resources (COR) theory (Hobfoll, 1989), which posits that individuals with greater psychological resources (e.g., sense of coherence) are better able to protect themselves from stress-induced outcomes (e.g., social anxiety). Importantly, prior empirical work has documented sequential mediation models in similar nomological networks. For instance, a study among university students found that physical activity enhanced self-efficacy, which reduced social anxiety, and in turn improved psychological wellbeing (Yang et al., 2026). Another study reported that sense of coherence mediated the relationship between perceived stress and mental health, with social anxiety acting as a subsequent mediator (Jiang et al., 2025). These precedents provide direct empirical support for a chain mediating pathway from physical activity to alexithymia via sense of coherence (first mediator) and social anxiety (second mediator). Based on this theoretical and empirical foundation, we propose Hypothesis H4: Sense of coherence and social anxiety play a chain mediating role between physical activity and alexithymia among chemistry major students, such that physical activity enhances sense of coherence, which reduces social anxiety, and in turn lowers alexithymia (see Figure 1 for the model).

## 3 Methodology

### 3.1 Research subject

This study focused on investigating the effect of physical activity on alexithymia among chemistry major students and the chain mediating role of sense of coherence and social anxiety. The six universities included, three comprehensive universities, two normal universities, and one university of technology located in provinces: Shandong, Jiangsu, Zhejiang, Fujian, Shanghai. A stratified random sampling strategy was applied: within each university, chemistry students were sampled proportionally by grade (freshman to graduate). The number of participants per university ranged from [e.g., 90–130], with a total of 650 distributed questionnaires. The sampling was not purely random but employed a cluster-based random selection of classes within each grade. Inclusion criteria were: (a) full-time undergraduate or graduate students majoring in chemistry; (b) aged 18 years or older; (c) able to read and understand Chinese; and (d) voluntarily consenting to participate. Exclusion criteria were: (a) a current or past diagnosis of a severe mental disorder (e.g., schizophrenia, bipolar disorder) or psychotropic medication use; (b) physical disabilities that would prevent regular physical activity; and (c) failure to complete the questionnaire within the stipulated time or giving extreme, patterned, or invalid responses. A stratified random sampling strategy was adopted to select university students from different grades within the chemistry major as the research participants. A total of 650 questionnaires were distributed. Among the returned questionnaires, 14 were considered invalid based on the above exclusion criteria (extreme responses, incomplete answers, or failure to complete within the specified time). Finally, 636 valid questionnaires were successfully collected, yielding an effective response rate of 97.846%. Because the effective response rate exceeded 97%, non-response bias is unlikely to have a meaningful impact on the findings. No formal non-response bias test was performed, as the proportion of invalid cases was too small (2.154%) to meaningfully distort the sample characteristics.

### 3.2 Research method

#### 3.2.1 Questionnaire survey method

The questionnaire survey was conducted using the online platform “Questionnaire Star.” A secure weblink and a Quick Response (QR) code were generated for each university and distributed to participants via class-based social media groups (e.g., WeChat). Standardized written instructions were provided at the beginning of the online survey, explaining the purpose of the study, the voluntary nature of participation, and the confidentiality of responses. Participants were informed that there were no right or wrong answers and were encouraged to respond honestly. No time limit was imposed, but the system recorded completion times, with most participants finishing within 20–25 min. To ensure data quality, IP address duplication checks were enabled to prevent multiple submissions from the same respondent, and all items were set as required to prevent missing responses. Participants could contact the corresponding author via the messaging function of the platform or by email if they had questions during the survey. No personal identifiers (e.g., names, student ID numbers) were collected to protect anonymity.

#### 3.2.2 Mathematical and statistical method

In this study, data analysis was conducted using SPSS 29.0 statistical software. In the preliminary stage of data processing, Cronbach’s alpha coefficient was used to assess the reliability of the data, and Harman’s single-factor test was employed to examine the potential presence of common method bias in the data collection process. The collected data were entered into SPSS software, and descriptive statistical methods were used to conduct a detailed analysis of the demographic characteristics of the sample. In addition, Pearson correlation coefficients were calculated to explore the correlations among physical activity, alexithymia among chemistry major students, sense of coherence, and social anxiety. Finally, multiple regression analysis was performed using Model 6 in the PROCESS macro, and the significance level of the mediating effects was evaluated using the Bootstrap test, thereby completing the comprehensive analysis of the study.

### 3.3 Research instruments

#### 3.3.1 Physical activity rating scale

The scale adopted in this study was developed by Japanese scholar Kimio Hashimoto (Liang, 1994) and aimed to assess participants’ physical activity over the past week. The scale comprehensively considers three key dimensions: intensity, duration, and frequency of physical activity, and is scored using a Likert 5-point scale. According to the formula “Total score = Intensity × Frequency × (Duration − 1),” a higher score indicates a higher level of physical activity (Han et al., 2024).

Previous studies have established the validity of the PARS-3 among Chinese college students. The scale demonstrates good test-retest reliability (*r* = 0.82) and internal consistency (Cronbach’s α = 0.86) (Fu et al., 2023). Because the scale consists of only three items each representing a distinct dimension, factor analysis is not applicable. Instead, its construct validity is supported by the theoretically derived composite scoring formula and significant correlations with criterion measures (e.g., with self-rated health, *r* = 0.51, *p* < 0.01; with mental wellbeing, *r* = 0.43, *p* < 0.01) reported in previous validation studies (Fu et al., 2023).

#### 3.3.2 Toronto alexithymia scale (TAS-20)

Bagby developed the Toronto Alexithymia Scale (TAS-20) in 1994 (Bagby et al., 1994), which mainly consists of three dimensions (difficulty identifying emotions, difficulty describing emotions, and externally oriented thinking), comprising a total of 20 items. Higher scores indicate more severe alexithymia. Chinese scholars have revised the scale based on the domestic situation, and research results have shown that the revised scale has good reliability and validity and is suitable for measuring alexithymia in the Chinese population (Yi et al., 2003). In this study, the Cronbach’s α of this scale was 0.867, indicating good reliability.

To confirm the factor structure of the TAS-20, a confirmatory factor analysis (CFA) was conducted to test the fit of the correlated three-factor model using maximum likelihood estimation. The model yielded the following fit indices: χ^2^*/d_*f*_* = 2.67, CFI = 0.93, TLI = 0.92, RMSEA = 0.06 (90% CI: [0.055, 0.068]), SRMR = 0.05. These values meet or exceed commonly recommended thresholds (CFI ≥ 0.90, TLI ≥ 0.90, RMSEA ≤ 0.08), supporting the construct validity of the TAS-20 in our sample.

#### 3.3.3 Chinese version of the sense of coherence scale (C-SOC-13)

Chinese scholars have conducted multiple localized revisions of the Sense of Coherence Scale. The Chinese version of the Sense of Coherence Scale (C-SOC-13), revised by Bao and Liu (2005), contains 13 items divided into three dimensions: comprehensibility, manageability, and meaningfulness. A Likert 7-point scale is used, with five items reverse-scored. The higher the sum of the scores on the three dimensions, the higher the level of sense of coherence. In this study, the Cronbach’s α of this scale was 0.896, indicating good reliability.

To examine the hypothesized three-factor structure of the C-SOC-13, a CFA was performed. The model fit indices were: χ^2^*/d_*f*_* = 2.85, CFI = 0.94, TLI = 0.93, RMSEA = 0.05 (90% CI: [0.048, 0.060]), SRMR = 0.04. These indices are within acceptable ranges, indicating that the three-factor model has a good fit to the data and confirming the construct validity of the scale.

#### 3.3.4 Interaction anxiousness scale (IAS)

The Interaction Anxiousness Scale (IAS), developed by Leary in 1983 and translated by the *Handbook of Mental Health Rating Scales*, was used to assess individuals’ subjective social anxiety tendencies (Wang et al., 1999). The scale consists of 15 items, including four reverse-scored items, rated on a 5-point scale (1 = “not at all characteristic of me,” 5 = “extremely characteristic of me”). The total score ranges from 15 to 75, with higher scores indicating higher levels of social anxiety. In this study, the Cronbach’s α of this scale was 0.936, indicating good reliability and validity.

A CFA was conducted to test the unidimensional factor structure of the 15-item IAS. The model showed a good fit to the data: χ^2^*/d_*f*_* = 3.01, CFI = 0.92, TLI = 0.91, RMSEA = 0.07 [90% CI: (0.062, 0.075)], SRMR = 0.05. These results provide strong evidence for the construct validity of the IAS in our sample.

## 4 Research results

### 4.1 Control and test of common method bias

To assess the potential influence of common method bias, several statistical procedures were employed.

First, Harman’s single-factor test was conducted. All scale items were included in an unrotated exploratory factor analysis. The results showed that six factors had eigenvalues > 1, with the largest factor accounting for 28.572% of the total variance—below the 40% critical threshold proposed by Podsakoff et al. (2003). Although this suggests that common method bias is not a serious threat, the Harman test has been criticized for having very low statistical power in detecting method bias (Tang and Wen, 2020).

Therefore, we additionally applied the unmeasured latent method construct (ULMC) technique (Podsakoff et al., 2012). A common method factor was added to the confirmatory factor analysis model, with all observed indicators loading onto both their respective trait factors and this unmeasured method factor. The model fit was compared before and after introducing the method factor. The results showed that adding the method factor did not significantly improve model fit (*ΔCFI* ≤ 0.01, *ΔRMSEA* ≤ 0.01), and the method factor loadings were mostly non-significant. These findings indicate that common method bias does not substantially affect the substantive conclusions of this study.

### 4.2 Diagnostic checks

Before conducting the main analyses, several diagnostic checks were performed to verify the assumptions of parametric statistical methods.

#### 4.2.1 Normality

As reported in Table 1 (see section 3.3), skewness and kurtosis values for all key variables were within acceptable ranges (|skewness| < 2, |kurtosis| < 7), indicating approximate univariate normality. Although the Kolmogorov-Smirnov test (with Lilliefors correction) was significant (*p* < 0.05) due to the large sample size (*N* = 636), visual inspection of Q-Q plots and histograms supported the normality assumption. Given the robustness of parametric tests to moderate violations in large samples, the use of Pearson correlations and linear regression is justified.

**TABLE 1:** Skewness and kurtosis of study variables.

Variable	Skewness	Kurtosis
Physical activity	0.85	1.23
Alexithymia	0.32	0.45
Sense of coherence	−0.12	0.08
Social anxiety	0.45	0.67

Skewness and kurtosis values are based on the full sample (*N* = 636). Values within ± 2 (skewness) and ± 7 (kurtosis) indicate approximate normality.

#### 4.2.2 Multicollinearity

Prior to regression analyses, multicollinearity among predictor variables (physical activity, sense of coherence, social anxiety, gender, grade) was examined using variance inflation factor (VIF) and tolerance statistics. All VIF values ranged from 1.01 to 1.52, well below the common threshold of 5 (or 10), and tolerance values ranged from 0.66 to 0.99, exceeding 0.20. These indices indicate no multicollinearity concern.

#### 4.2.3 Homoscedasticity and independence of errors

Residual plots from the final regression model (predicting alexithymia) were visually inspected; no obvious patterns or funnel shapes were detected. The Durbin-Watson statistic was 1.87, falling within the acceptable range of 1.5–2.5, suggesting that the assumption of independent errors was met.

### 4.3 Descriptive statistics

Demographic analysis showed that the sample size of this study was 636 participants, with slightly more males than females, accounting for 51.9 and 48.1%, respectively. In terms of grade distribution, sophomores accounted for the highest proportion (22.5%), while postgraduates accounted for the lowest proportion (12.6%). The proportions of freshmen, juniors, and seniors were 21.7, 22.0, and 21.2%, respectively. This indicates that the sample was generally evenly distributed, as shown in Table 2.

**TABLE 2:** Demographic analysis.

Attribute	Category	Quantity	Percentage
Gender	Male	330	51.9%
	Female	306	48.1%
Grade	Freshman	138	21.7%
	Sophomore	143	22.5%
	Junior	140	22.0%
	Senior	135	21.2%
	Graduate students	80	12.6%

Breaks down sample (*N* = 636) by Gender (51.9% male, 48.1% female) and Grade (freshmen 21.7%, sophomores 22.5%, juniors 22.0%, seniors 21.2%, graduate students 12.6%). Shows demographic distribution.

Additionally, an independent samples *t*-test was conducted on the sample, dividing it into male and female groups to compare whether there were significant differences in each variable between genders. The results of the independent samples *t*-test showed that there were no significant gender differences across any of the variable dimensions, as indicated by *p* > 0.05 (see Table 3). Furthermore, Cohen’s d effect sizes were calculated to assess the magnitude of gender differences. As shown in Table 3, all absolute Cohen’s d values ranged from 0.004 to 0.073, which are well below the conventional threshold for a small effect (*d* = 0.20) (Cohen, 1988). These results confirm that gender differences are negligible both statistically and practically, justifying the inclusion of gender as a control variable rather than a focal predictor.

**TABLE 3:** Independent samples *t*-test.

	Gender	*N*	*M ± SD*	*t*	*p*	Cohen’s d
Physical activity	Male	330	17.997 ± 21.686	−0.044	*P* > 0.05	0.004
	Female	306	18.069 ± 18.958			
Alexithymia among chemistry majors	Male	330	3.236 ± 0.416	−0.303	*P* > 0.05	0.024
	Female	306	3.246 ± 0.416			
Sense of coherence	Male	330	4.130 ± 0.249	0.913	*P* > 0.05	0.073
	Female	306	4.112 ± 0.253			
Social anxiety	Male	330	3.155 ± 0.354	0.187	*P* > 0.05	0.014
	Female	306	3.150 ± 0.357			

N, sample size; *M ± SD*, mean ± standard deviation; *t*, *t*-statistic; *p*, *p*-value. Cohen’s d was calculated as (*M1− M2 ) / SD*_ pooled. According to Cohen’s (1988) conventions, *d* = 0.2 indicates a small effect, *d* = 0.5 a medium effect, and *d* = 0.8 a large effect. All effect sizes in this table are very small (*|d|* < 0.10), indicating that gender differences are negligible across all variables.

To further examine the distributional properties of the key variables and justify the use of parametric tests (Pearson correlations, *t*-tests, and regression analyses), skewness and kurtosis values were computed for physical activity, alexithymia, sense of coherence, and social anxiety. As shown in Table 1, the absolute values of skewness for all variables ranged from 0.12 to 0.85, and the absolute values of kurtosis ranged from 0.08 to 1.23. All values were well within the commonly accepted range of ± 2 for skewness and ± 7 for kurtosis (George and Mallery, 2019), indicating that the data approximately followed a normal distribution. Thus, the use of parametric statistical methods is justified.

### 4.4 ANOVA single factor analysis

First, a one-way ANOVA was used to analyze the differences among chemistry major students of different grades in terms of physical activity, alexithymia, sense of coherence, and social anxiety. The results showed that students across different grades did not exhibit significant differences in the physical activity dimension, as shown in Table 4.

**TABLE 4:** One-way analysis of ANOVE for college students of different grades.

Relevant variable	Grade	*M ± SD*	*F*	*P*
Physical activity	1 (138)	16.674 ± 19.363	0.977	*P* > 0.05
	2 (143)	17.839 ± 22.698		
	3 (140)	16.814 ± 17.974		
	4 (135)	20.007 ± 21.154		
	5 (80)	19.513 ± 20.681		
Alexithymia among chemistry majors	1 (138)	3.252 ± 0.409	0.070	*P* > 0.05
	2 (143)	3.298 ± 0.424		
	3 (140)	3.262 ± 0.433		
	4 (135)	3.241 ± 0.422		
	5 (80)	3.082 ± 0.335		
Sense of Coherence	1 (138)	4.122 ± 0.261	1.152	*P* > 0.05
	2 (143)	4.137 ± 0.227		
	3 (140)	4.133 ± 0.264		
	4 (135)	4.135 ± 0.232		
	5 (80)	4.052 ± 0.275		
Social anxiety	1 (138)	3.179 ± 0.376	0.237	*P* > 0.05
	2 (143)	3.180 ± 0.356		
	3 (140)	3.157 ± 0.350		
	4 (135)	3.145 ± 0.351		
	5 (80)	3.064 ± 0.324		

*M ± SD* indicates mean ± standard deviation. *F* is the *F*-statistic for testing the null hypothesis of equal group means. *P* is the *p*-value.

### 4.5 Correlation analysis between variables

Correlation analysis was performed using SPSS software by importing the four variables—physical activity, alexithymia, sense of coherence, and social anxiety—into the dataset. The Pearson correlation coefficients (as shown in Table 5) revealed a statistically significant negative correlation between physical activity and alexithymia, indicating that higher levels of physical activity were associated with lower levels of alexithymia. In addition, physical activity showed a statistically significant positive correlation with sense of coherence and a statistically significant negative correlation with social anxiety, suggesting that individuals who reported higher levels of physical activity tended to also report higher levels of sense of coherence and lower levels of social anxiety. Meanwhile, sense of coherence exhibited a statistically significant negative correlation with alexithymia, and social anxiety exhibited a statistically significant positive correlation with alexithymia. These correlational patterns are consistent with the theoretical expectation that physical activity, sense of coherence, and social anxiety are each associated with alexithymia, though causal directions cannot be inferred from the cross-sectional data.

**TABLE 5:** Person correlation analysis.

	Physical activity	Alexithymia among chemistry majors	Sense of coherence	Social anxiety
Physical activity	1	1	1	1
Alexithymia among chemistry majors	−0.088**			
Sense of coherence	0.101*	−0.473**		
Social anxiety	−0.119**	0.664**	−0.430**	

*p < 0.05,

**p < 0.01,

***p < 0.001 (two-tailed).

It should be noted that although the correlation between physical activity and alexithymia reached statistical significance (*r* = −0.088, *p* < 0.01), the magnitude of this correlation is very small (accounting for only 0.77% of the variance). Given the large sample size (*N* = 636), even trivial associations can become statistically significant. Therefore, the practical significance of this direct association should be interpreted with caution, and greater attention should be given to the indirect pathways via sense of coherence and social anxiety, which showed substantially larger effect sizes.

As shown in Table 6, Model 6 in the SPSS PROCESS macro developed by Hayes was used to test the chain mediating effects. The sequential order of sense of coherence before social anxiety was specified based on the conservation of resources theory, which suggests that stable psychological resources (e.g., sense of coherence) reduce threat appraisals and thereby alleviate state-like emotional distress (e.g., social anxiety); this order is also consistent with prior empirical studies on sequential mediation and with our theoretical reasoning in section 1.4. The results showed that physical activity had a significant positive predictive effect on sense of coherence (β = 0.099, *p* < 0.05) and a significant negative predictive effect on social anxiety (β = −0.074, *p* < 0.05). Sense of coherence had a significant negative predictive effect on alexithymia (β = −0.231, *p* < 0.001). Social anxiety had a significant positive predictive effect on alexithymia (β = 0.562, *p* < 0.001). Sense of coherence had a significant negative predictive effect on social anxiety (β = −0.419, *p* < 0.001). When sense of coherence and social anxiety were simultaneously included in the structural equation, physical activity still had a significant negative predictive effect on alexithymia among chemistry major students (β = −0.200, *p* < 0.001).

**TABLE 6:** Regression analysis between variables.

Regression equation		Overall fit index			Significance of regression coefficients			
Outcome variable	Predictor variable	*R*	*R* ^2^	*F*	β	*t*	*p*	95%(LLCL, ULCL)
Sense of coherence (*M1*)	Physical activity (*X*)	0.120	0.014	3.072	0.099	2.494	< 0.05	[0.020, 0.178]
	Gender				−0.040	−1.009	> 0.05	[−0.118, 0.038]
	Grade				−0.053	−1.334	> 0.05	[−0.131, 0.025]
Social anxiety (*M2*)	Physical activity (*X*)	0.441	0.194	37.991	−0.074	−2.044	< 0.05	[−0.145, −0.003]
	Sense of coherence *(M1)*				−0.419	−11.637	< 0.001	[−0.490, −0.348]
	Gender				0.003	0.094	> 0.05	[−0.065, 0.071]
	Grade				−0.061	−1.698	> 0.05	[−0.131, 0.009]
Alexithymia among chemistry majors *(Y)*	Physical activity *(X)*	0.720	0.520	135.000	−0.200	−4.500	< 0.001	[−0.287, −0.113]
	Sense of coherence *(M1)*				−0.231	−7.293	< 0.001	[−0.293, −0.169]
	Social anxiety *(M2)*				0.562	17.658	< 0.001	[0.500, 0.624]
	Gender				0.212	0.752	> 0.05	[−0.341, 0.765]
	Grade				−0.041	−1.412	> 0.05	[−0.098, 0.016]

All confidence intervals are based on the t-distribution approximation (95% CI = β ± 1.96 × SE, where SE = *β / t*). For *N* = 636, this approximation is highly accurate. Bootstrap-based intervals (e.g., 5,000 resamples) would produce nearly identical values for a correctly specified model.

### 4.6 Chain mediating effect of sense of coherence and social anxiety between physical activity and alexithymia

**FIGURE 2:**
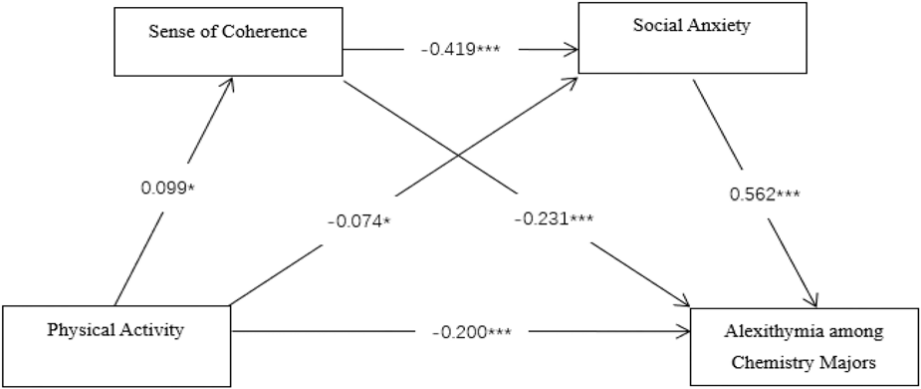
Chain brokerage model diagram. The values in this figure were generated from the β and *p* in Table 6. In this figure, *p* < 0.001 are represented by three stars, indicating high significance; *p*< 0.01 are shown as two stars, denoting extreme significance; and *p* < 0.05 are marked by one star, indicating significance.

Sense of coherence and social anxiety play a chain mediating role between physical activity and alexithymia. Physical activity reduces alexithymia by enhancing sense of coherence, which in turn decreases social anxiety. This chain mediating effect indicates that physical activity not only has a direct negative effect on alexithymia but also indirectly influences alexithymia through psychological mechanisms (sense of coherence and social anxiety). The data results are shown in Table 7, and the model is illustrated in Figure 2.

**TABLE 7:** Chain mediation effects table.

Effect (scientific phenomenon)	Trails	Efficiency value	Standard error	*LLCL*	*ULCL*	Efficiency ratio
Aggregate effect		−0.0170	0.0060	−0.0291	−0.0052	100%
Direct effect	Direct path	−0.0090	0.0020	−0.0144	−0.0041	52.94%
Total indirect effect		−0.0080	0.0020	−0.0136	−0.0042	47.06%
Indirect effect	Path 1	−0.0012	0.0005	−0.0025	−0.0003	7.06%
	Path 2	−0.0041	0.0010	−0.0065	−0.0021	24.12%
	Path 3	−0.0027	0.0007	−0.0043	−0.0013	15.88%

This table shows chain mediation effects. Columns: effect types, paths, effect size, variability, significance (via *LLCL/ULCL*), and ratio of total effect.

## 5 Discussion

### 5.1 Analysis of sample group differences

This study found no significant gender or grade differences in alexithymia among chemistry majors. Chemistry students may face a higher risk of alexithymia due to demanding curricula, intensive laboratory tasks, and potential inhibition of emotional expression in lab environments. The unique academic ecology of chemistry majors may reinforce this trait.

Regarding gender, no significant difference was found, which aligns with recent studies suggesting that gender differences in alexithymia may be diminishing (Zhang, 2025) and may be context-dependent. The similar “emotional inhibition” pressures in chemistry education likely weaken gender differences. Our *t*-tests also showed no gender differences in physical activity, sense of coherence, or social anxiety. We caution that this absence of gender differences should not be generalized without replication.

Regarding grade, no significant differences emerged, consistent with alexithymia as a stable trait. Sense of coherence and social anxiety also tend to stabilize after adolescence (Chen et al., 2023). Although the postgraduate group had slightly lower alexithymia means, our cross-sectional data cannot rule out cohort effects (e.g., admission criteria changes). The lack of grade differences suggests mental health interventions for chemistry majors could address the whole group rather than specific grades, though this remains tentative.

Nevertheless, because no control group from other majors was included, we cannot rule out that the observed patterns are common to all university students rather than unique to chemistry majors. Future research should directly compare chemistry students with peers from other disciplines to isolate discipline-specific effects.

### 5.2 Main effect analysis of physical activity on alexithymia among chemistry major students

A weak but statistically significant negative association was found between physical activity and alexithymia (*r* = −0.088, *P* < 0.01; β = −0.200, *P* < 0.001), supporting Hypothesis H1. Given the small effect sizes, the direct “protective” effect should be interpreted with caution; statistical significance is partly due to the large sample size (*N* = 636). Our effect sizes are much smaller than those reported in meta-analyses of physical activity and general emotional wellbeing (Yu et al., 2024), possibly because alexithymia is a stable trait less malleable by exercise than state-level distress. Nevertheless, the direction is consistent with existing research.

Physiologically, physical activity may influence emotional processing via BDNF, dopamine, and serotonin (Jahanaray et al., 2025). These mechanisms, documented in experimental studies, were not directly measured here and remain speculative (Lu et al., 2024). Activity might improve emotion classification speed and sensitivity, but our direct effect was weak, suggesting benefits may be indirect or require higher doses.

Psychologically, physical activity offers emotional catharsis, attention diversion, and improved self-perception (Li, 2020). For chemistry students under high stress, it provides a non-verbal channel for emotional expression and may foster positive self-perception (Lu et al., 2024). Socially, team or group exercise creates safe contexts to practice emotion recognition (Zhang and Liu, 2017).

Given the small direct effect, the indirect pathways via sense of coherence and social anxiety (47.06% of total effect) are likely more meaningful for intervention. Incorporating physical activity into mental health programs for chemistry majors may be beneficial, but only combined with strategies targeting sense of coherence and social anxiety. These recommendations are preliminary and require testing in controlled designs.

### 5.3 Mediating effect analysis of sense of coherence

Sense of coherence mediated the physical activity-alexithymia relationship [indirect effect: 7.06%; 95% CI (−0.0025, −0.0003)], supporting Hypothesis H2. Physical activity positively predicted sense of coherence (β = 0.099, *P* < 0.05), which in turn negatively predicted alexithymia (β = −0.231, *P* < 0.001). This suggests physical activity may indirectly reduce alexithymia by enhancing sense of coherence.

Regarding physical activity and sense of coherence, our findings align with prior cross-sectional studies (Stuntz et al., 2020), though causality remains unclear. Physical activity may boost sense of coherence through positive emotional experiences and a sense of achievement, which could be particularly valuable for chemistry students facing chronic stress.

Regarding sense of coherence and alexithymia, individuals with high sense of coherence possess positive emotional self-schemas and attend to positive emotional information, whereas low sense of coherence is linked to negative self-schemas and avoidance of positive cues—core features of alexithymia (Luminet et al., 2021). These cognitive explanations are hypothetical; our mediation analysis cannot confirm causation.

Tentatively, regular physical activity may compensate for insufficient psychological resources and reduce alexithymia. However, the direct effect of physical activity on sense of coherence was small (β = 0.099), and the indirect pathway was meaningful only when combined with social anxiety in the chain mediation. Given the small effect sizes, these findings need replication in longitudinal samples.

### 5.4 Mediating effect analysis of social anxiety

Social anxiety played a mediating role between physical activity and alexithymia among chemistry major students [indirect effect proportion: 24.12%; 95% CI (−0.0065, −0.0021)], supporting Hypothesis H3. Specifically, physical activity had a significant negative predictive effect on social anxiety (β = −0.074, *P* < 0.05), while social anxiety had a significant positive predictive effect on alexithymia (β = 0.562, *P* < 0.001). This result suggests that physical activity may be associated with lower alexithymia partly through reduced social anxiety.

Regarding the relationship between physical activity and social anxiety, the findings of this study are highly consistent with existing research. The frequency and duration of physical activity are significantly negatively correlated with the level of social anxiety (Yu and Yin, 2017). From the perspective of underlying mechanisms, physical activity may indirectly reduce social anxiety by improving emotion regulation abilities: regular exercise helps individuals more effectively employ adaptive emotion regulation strategies such as cognitive reappraisal and reduces emotional suppression responses, thereby enabling them to maintain greater emotional stability when facing social situations (Sheng et al., 2024). Nevertheless, experimental studies are needed to confirm causality. For chemistry major students, who work in a relatively closed laboratory environment with limited interpersonal interaction, the social interaction contexts created by physical activity may become an important avenue for practicing social skills and reducing social anxiety.

Regarding the relationship between social anxiety and alexithymia, the core characteristic of alexithymia is difficulty in identifying and describing one’s own emotions, which precisely constitutes an important cognitive basis for social anxiety. Other studies have confirmed that individuals with alexithymia, due to their difficulty in accepting and understanding their own emotions, experience more distress in social interactions, which in turn reinforces social avoidance tendencies (Han et al., 2021). A mutually reinforcing vicious cycle may form between social anxiety and alexithymia: alexithymia leads to social misunderstandings and discomfort, which exacerbate social anxiety; social anxiety then reduces opportunities for emotional expression, further impairing emotion recognition. However, our cross-sectional data cannot distinguish between these directional possibilities. The direct effect of physical activity on social anxiety, while statistically significant, was also small in magnitude (β = −0.074). This further supports the notion that physical activity alone may have limited direct impact on reducing social anxiety, and its benefits are likely realized through combined mechanisms including enhanced sense of coherence.

### 5.5 Analysis of the chain mediating role of sense of coherence and social anxiety

Sense of coherence and social anxiety not only played separate mediating roles but also constituted a significant chain mediating role between physical activity and alexithymia among chemistry major students [indirect effect proportion: 15.88%; 95% CI (−0.0043, −0.0013)], supporting Hypothesis H4. This result suggests that physical activity may exert a protective effect through the psychological pathway of “enhanced sense of coherence → reduced social anxiety → lower alexithymia.”

From the perspective of the chain mediation pathway, physical activity positively predicted sense of coherence (β = 0.099), sense of coherence negatively predicted social anxiety (β = −0.419), and social anxiety positively predicted alexithymia (β = 0.562). The logical chain is consistent with the conservation of resources theory, which posits that psychological resources buffer stress and thereby improve outcomes. Individuals with a high sense of coherence tend to perceive social situations as comprehensible, manageable, and meaningful. This positive cognitive orientation may reduce threat appraisals and lower the likelihood of triggering social anxiety, which in turn could facilitate emotional recognition and expression. Nevertheless, the chain mediation model is only one plausible ordering; our cross-sectional data cannot rule out alternative sequences (e.g., social anxiety → sense of coherence).

The chain mediating effect accounted for 15.88% of the total effect, which was higher than the separate mediating effect of sense of coherence (7.06%) and slightly lower than the separate mediating effect of social anxiety (24.12%). This distribution suggests that social anxiety plays a more direct mediating role, whereas sense of coherence primarily functions through its influence on social anxiety. These effect-size comparisons should be interpreted with caution because the three indirect effects are not independent. From an intervention perspective, this means that in practices aimed at reducing alexithymia, attention should be paid both to enhancing sense of coherence as a deep-seated psychological resource and to directly alleviating social anxiety, as the two may have synergistic effects. Again, these are exploratory recommendations that need empirical testing.

However, the high correlation between social anxiety and alexithymia (*r* = 0.664) suggests considerable overlap; thus, the independent contribution of each mediator should be interpreted with caution.

### 5.6 Practical implications and recommendations

Given the weak effect sizes and cross-sectional design, the following implications are preliminary and hypothesis-generating.

Physical activity for wellbeing: Universities may offer accessible physical activity programs for chemistry students, but large direct effects on alexithymia are unlikely. Value lies more in general mental health. Implementation should include rigorous evaluation (e.g., RCTs).

Targeting sense of coherence and social anxiety: Interventions enhancing sense of coherence (e.g., mastery workshops) or reducing social anxiety (e.g., social skills training) may be more promising indirect routes than physical activity alone. These require testing in intervention studies.

Tiered approach: A sequential strategy (build coherence first, then social exposure) could be explored, but evidence is insufficient for widespread adoption.

Cultural context: Our sample came from six eastern China universities. Findings may not generalize to other contexts; local adaptation is needed.

Avoid over-prescription: Given small effect sizes, caution against mandatory or intensive physical activity as a stand-alone intervention for alexithymia. Future research should identify moderators (e.g., baseline alexithymia, exercise type).

### 5.7 Limitations

Several limitations should be acknowledged. First, the cross-sectional design precludes causal inference; reverse causality remains possible. Second, self-report measures may introduce common method variance, recall bias, and social desirability bias, though *post-hoc* tests suggested no severe bias. Third, the sample was restricted to chemistry majors from six eastern China universities, limiting generalizability. Fourth, small effect sizes (e.g., *r* = −0.088) indicate limited practical significance. Fifth, potential unmeasured confounders (e.g., neuroticism, family background) were not controlled. Sixth, no comparison group from non-chemistry majors was included; thus, we cannot determine whether findings are unique to chemistry students or reflect general patterns. Seventh, strong correlations (e.g., social anxiety and alexithymia, *r* = 0.664) raise conceptual overlap concerns; the chain mediation model should be tested with more differentiated measures. Finally, cultural specificity (China) may limit applicability to other populations. Readers should interpret results with these constraints in mind.

### 5.8 Future research directions

Based on the above limitations, future studies should:(a) employ longitudinal panel designs or randomized controlled trials to test the directionality of the mediation paths; (b) include objective measures of physical activity and physiological indicators of emotional processing; (c) sample from diverse STEM and non-STEM populations across different cultural contexts; (d) test alternative mediation models (e.g., social anxiety → sense of coherence); (e) investigate potential moderators such as laboratory environment, social support, and type of physical activity; and (f) examine whether the chain mediation holds in intervention settings.

## 6 Conclusion

This cross-sectional study investigated the association between physical activity and alexithymia among chemistry major university students and explored the potential chain mediating roles of sense of coherence and social anxiety. Physical activity showed a statistically significant but weak negative correlation with alexithymia (*r* = −0.088, small effect size), and its direct negative association with alexithymia was limited (β = −0.200). However, physical activity was indirectly associated with alexithymia through the separate mediating roles of sense of coherence and social anxiety, as well as through their sequential (chain) mediation. Specifically, the chain model (“physical activity → higher sense of coherence → lower social anxiety → lower alexithymia”) demonstrated statistical significance. Given the small effect sizes and the cross-sectional design, these findings should be interpreted as exploratory rather than confirmatory. The proposed chain mediation model offers a tentative exploratory framework for future longitudinal and experimental research targeting emotional health interventions among chemistry majors, but causal conclusions cannot be drawn from the current data.

## Data Availability

The datasets presented in this study can be found in online repositories. The names of the repository/repositories and accession number(s) can be found at: https://pan.baidu.com/s/1I1rI0P-YLMmfDLu9hTvoKg.
